# Vertebroplasty combined with facet joint block vs. vertebroplasty alone in relieving acute pain of osteoporotic vertebral compression fracture: a randomized controlled clinical trial

**DOI:** 10.1186/s12891-022-05753-4

**Published:** 2022-08-23

**Authors:** Sha-Jie Dang, Wen-Bo Wei, Ling Wei, Jin Xu

**Affiliations:** 1 The Key Laboratory of Biomedical Information Engineering of Ministry of Education, Institute of Health and Rehabilitation Science, School of Life Science and Technology, Xi’an Jiaotong University, Xi’an, 710049 China; 2 Department of Anesthesia, Shaanxi Provincial Cancer Hospital, Xi’an, 710061 China; 3 Department of Orthopedics, Shaanxi Provincial people’s Hospital, Xi’an, 710068 China; 4 Department of Pain, YangLing Demonstration Zone Hospital, Yang ling, 712100 China

**Keywords:** Osteoporotic vertebral compression fractures, Facet joint block, Percutaneous vertebroplasty

## Abstract

**Objective:**

The study objective was to compare the efficacy and safety of percutaneous vertebroplasty (PVP) combined with facet joint block (FB) and vertebroplasty alone in relieving acute pain on osteoporotic vertebral compression fractures (OVCFs).

**Methods:**

A prospective, randomized controlled study was conducted. One hundred ninety-eight patients of OVCFs undergoing surgery were randomly divided into two groups: Group P (PVP, *n* = 97), Group PF (PVP + FB, *n* = 101). The Visual analogue scale (VAS) and Oswestry disability index (ODI) were measured during pre-operation, 1 day, 1, 3, 6 and 12 months after the operation, respectively. The hospitalization time, operation time, complications, recurrence, the mean amount of cement injected and the number of patients who applied Cox-2 inhibitors within 3 days after operation were compared in the two groups.

**Results:**

The VAS and ODI scores at each observation point of the post-operation were significantly decreased than that at the pre-operation in both groups (*P* < 0.05). The VAS and ODI scores in Group PF were significantly lower than that in Group P 1 day and 1 month after the operation (*P* < 0.05). The number of patients who applied Cox-2 inhibitors within 3 days after operation in group PF was significantly lower that in Group P (*P* < 0.001). There was no significant difference in hospitalization time, operation time, the mean amount of cement injected, complication rate, VAS and ODI scores at the pre-operation (*P* > 0.05).

**Conclusion:**

Both PVP combined with FB and PVP alone are effective treatment methods for OVCFs. But PVP combined with FB showed better back pain relief than PVP alone in the short term after the operation for OVCFs.

## Introduction

With the increase of the elderly population, the incidence of osteoporotic vertebral compression fractures (OVCFs) is increasing rapidly [1]. In the elderly population, OVCFs commonly cause severe back pain, substantial vertebral deformity, disturbances in activities of daily living, decreased quality of life and increased adjacent spinal fractures and mortality [2]. Many studies report that percutaneous vertebroplasty (PVP), which injects the polymethylmethacrylate (PMMA) into the fractured vertebral body, as minimally invasive surgery, has the advantages of the shorter operation time, less trauma and significant pain relief [3]. It is considered the preferred method for the treatment of osteoporotic vertebral compression fractures. However, the effectiveness of the surgery is still a controversial topic. The percentage of patients who experienced unsatisfactory back pain relief after PVP ranged from 5 to 22% [4]. The causes of low back pain caused by OVCFs are complex. The pain associated with OVCFs may not only come from the vertebral body but also the posterior elements [5, 6]. Therefore, facet joint block (FB) which can eliminate pain originating from the posterior facet joint would be beneficial for alleviating acute back pain associated with OVCFs [7]. But FB can’t restore vertebral height or reverse kyphotic deformity. We consider that PVP combined with FB can both reduce the pain from vertebral body and the posterior elements. At present, there are few studies on PVP combined with facet block. Is the PVP combined with FB an effective solution for alleviating the acute pain caused by OVCFs and reduce residual pain after PVP?

Thus, the purpose of this study was to compare the clinical effect of PVP combined with FB and PVP alone in the treatment of pain of the OVCFs, and evaluate the effectiveness and safety.

## Methods

### Study design

A prospective, randomized controlled study was conducted at the departments of Orthopedic Surgery in Shannxi Provincial People’s Hospital between January 2018 and December 2020. Written informed consent was obtained from each of the patient, otherwise their next of kin or their legal representative. This study was approved by the clinical research ethics committee of Shannxi Provincial People’s Hospital (no. 2018–039), and was registered in Chinese Clinical Trail Registry (registration number ChiCTR-IOR-2200056526). This study followed the Good Clinical Practice guidelines and the guidelines of the Helsinki Declaration.

### Patients

In this study, we screened patients who received PVP or PVP combined with FB surgery for OVCFs. The inclusion criteria were as follow: 1) single-level fresh lumbar OVCFs, 2) older than 65 years, 3) with the back pain less than 6 weeks, and ineffective to medical therapy, 4) the visual analog scale (VAS) pain score was 7 or higher, 5) bone mineral density (BMD) T-scores less than − 2.5, 6) spinal MRI scan showed bone marrow edema of the affected vertebrae, 7) the posterior wall of the vertebral body remaining intact without any neurologic deficit or compression in the spinal canal, 8) the patient willing to receive PVP treatment with or without an FB. The exclusion criteria were as follow: 1) infection, 2) radicular and/or cord compression syndrome, 3) patients who are unable to operate due to mental or organ dysfunction, 4) burst vertebral fracture with spinal canal stenosis and neurologic deficit, 5) spinal infection or skin disease, 6) previous lumbar surgery, 7) patients who are lost to follow-up.

### Randomization and masking

A total of 220 patients with OVCFs were enrolled in this study. A biostatistician, who was independent of data management and statistical analyses, generated random numbers (in a 1:1 ratio) using the SAS 9.2 software (SAS Institute, Cary, NC). The results of randomization were stored online (https://pan.baidu.com) until the end of the study. Surgeons logged in online and selected groups according to the randomization order. Throughout the study, researchers, health-care team members, and patients were masked to the group’s assignment. And the collection of case information, postoperative follow-up, and statistical analysis were performed by investigators blinded to the group of patients. In an emergency, unmasking of the treatment allocation could be requested, and the study would be terminated. Finally, a total of 198 patients with OVCFs were randomly divided into two groups: Group P (PVP) (*n* = 97), Group PF (PVP + FB) (*n* = 101).

### Procedures

U-shaped pillows are under the patient’s chest and ilium to make the patient’s abdomen is suspended. Guided by C-arm fluoroscopy, the patient is placed in the prone positions. The injection site was sterilized with antiseptic fluid and draped with surgical towels. Both PVP and FB were performed by spine surgeons in our department.

In the PVP procedure, PVP was performed by bilateral or unilateral transpedicular approach. After satisfactory local anesthesia, the puncture needle was inserted into the fractured vertebral body through the pedicle. Fluoroscopy showed that the puncture needle was in a proper position. Under fluoroscopy, 3–6 ml polymethylmethacrylate (PMMA) was injected into the fractured vertebral body to ensure full filling and avoid pulmonary embolism or intraspinal leakage due to bone cement leakage.

Satisfactory cement distribution was defined as cement spread from the superior to the inferior end plate, from the medial cortex of the pedicle to the medial cortex of the contralateral pedicle, and from the anterior cortex of the vertebral body to the posterior third of the vertebral body. Stop the operation when the bone cement spreads to the posterior third of the vertebral body, which can avoid the bone cement from leaking into the spinal canal. The amount of bone cement injected was 1.5 ~ 6.0 ml.

**Fig. 1:**
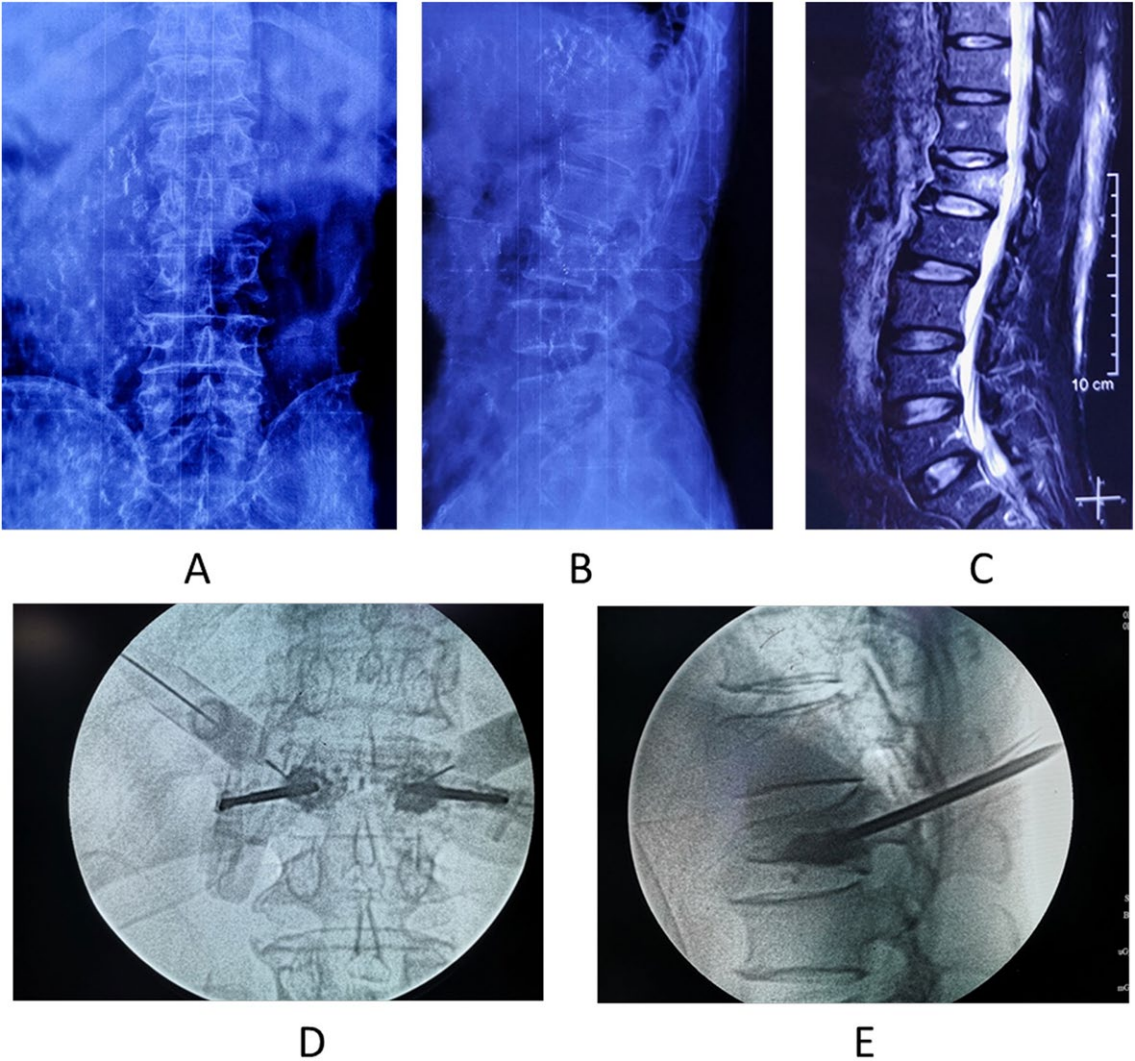
**A B** Anteroposterior and Lateral view shows the compressed L1 vertebra. **C** Short tau inversion recovery sequences magnetic resonance image. **D E** Anteroposterior and Lateral view shows the needle was inserted the juncture of the superior articular process and transverse process

In the FB procedure, under the guidance of a fluoroscope, a no. 23 gauge needle was inserted. The facet joint of the same vertebra was blocked. The target was the juncture of the superior articular process and transverse process for L1–4 levels and at the junction of the superior articular process and the top border of the sacral crest for the L5 level. Then the needle was slightly retracted from its intraarticular location. Because each facet joint receives a double innervation. A needle was first placed into the facet joint space. Then the needle was slightly retracted from its intraarticular location and placed onto the surface of the facet joint capsule, the medial and lateral margins of the facet joints,both inferior and superior facet joints, should be included in the process of capsular admixture infiltration under fluoroscopic guidance. Confirmation of the position of the needle with the AP and lateral images acquired using fluoroscopy. The mixture solution was composed of 80 mg methylprednisolone, 10 mL 2% lidocaine and 5 mL 1% ropivacaine，then 2 ml of mixture solution was injected around the facet joint (Fig. 1).

After the operation, the patient was given standardized anti-osteoporosis treatment (Calcium carbonate 600 mg and Calcitriol 0.25 μg were administered daily), and the patient was advised to wear a brace for functional exercise within 3 months. Cox-2 inhibitors (oral Celecoxib, 200 mg, bid) would be given as required if patients had surgical site pain (VAS > 3) within 3 days after the operation.

Gender, Age, BMI, Bone Mineral Density (BMD), operating time, the amount of bone cement injected, hospitalization time, complications, recurrence and the number of patients who applied Cox-2 inhibitors within 3 days after operation were recorded. The visual analog scale (VAS) and the Oswestry Disability Index (ODI) scores were measured during pre-operation, 1 day, 1, 3, 6 and 12 months after the operation, respectively. The VAS score was measured using a 10 cm visual analog scale. It was evaluated from 0 to 10, 0 indicates no pain, and 10 indicates the most severe pain. The ODI assesses low back pain-related disability, the higher the score means the worse the disability.

### Outcomes

The primary outcomes were VAS and ODI score pre-operation, 1 day, 1, 3, 6, and 12 months after the operation. The secondary outcomes included operation time, hospitalization time, complication, recurrence and the number of patients who applied Cox-2 inhibitors within 3 days after operation.

### Statistical analysis

The primary endpoint was the VAS 1 day and 1 month after the operation. In the preliminary study, 20 patients were assigned to Group P and Group PF (*n* = 10), and a sample size of 95 per group was obtained by PASS 11.0 (NCSS, LLC, Kaysville, Utah, USA) with two-tailed α = 0.05 and β = 0.90. Take into account a dropout rate of approximately 10–20%, we planned to enroll 110 patients for each group.

The statistical analysis was performed using SPSS 24.0 (SPSS, Inc., IBM). Numeric variable was expressed as Mean ± SD and categorical data was expressed by N (%). Numeric variable was analyzed by t-test and categorical data was analyzed with the χ2 test. The VAS and ODI scores at the different time were analyzed by a repeated measure analysis of variance (ANOVA), and Bonferroni’s correction was used for post hoc analysis. The value of *P* < 0.05 is treated as significant differences.

## Result

### General information

**Fig. 2:**
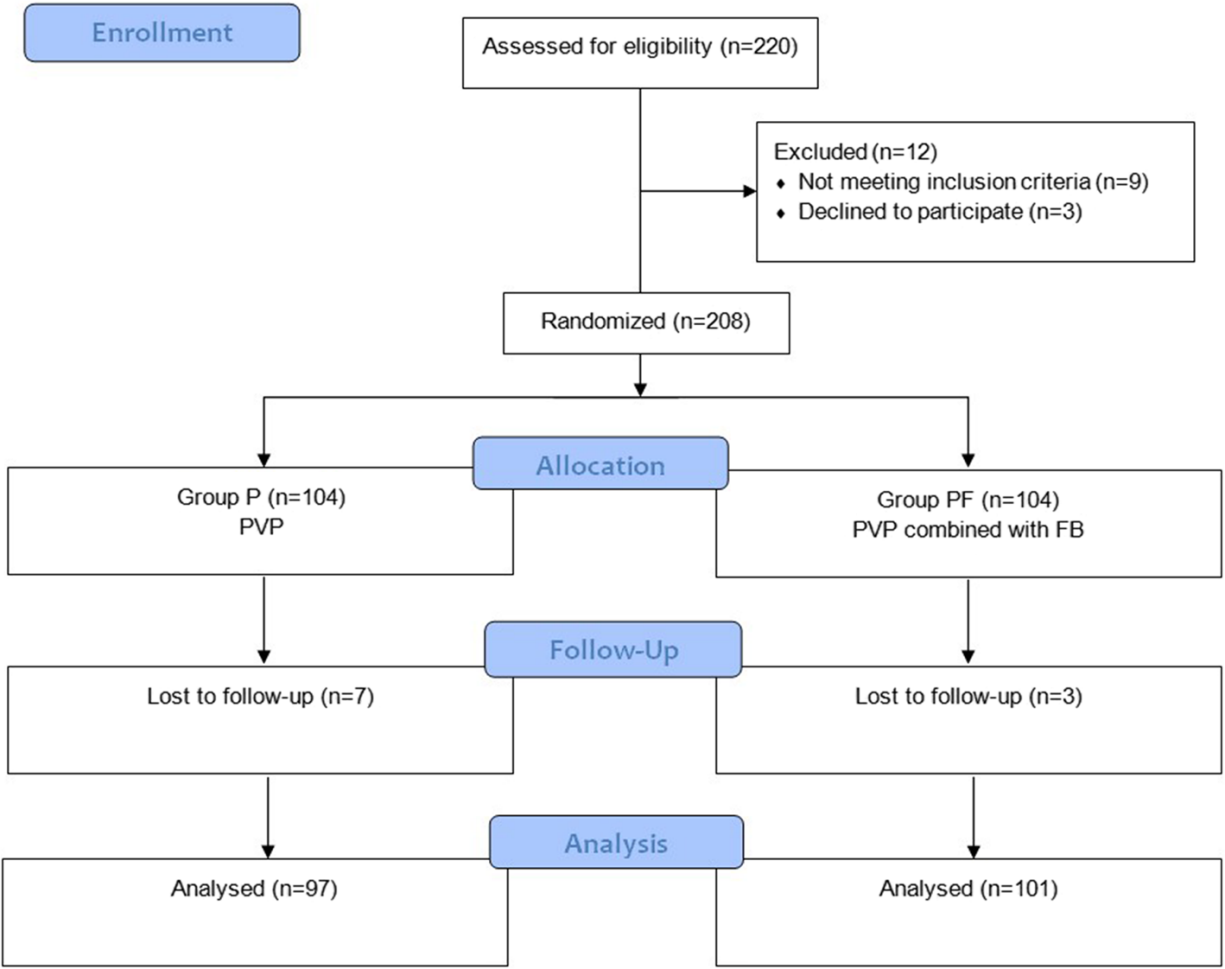
Consolidated Standards of Reporting Trials (CONSORT) flow diagram

A total of 220 patients were enrolled in this study. Nine patients were excluded from the study due not meeting inclusion criteria. Three patientsrefused to participate before surgery. Seven patients were excluded because oflosting to follow-up in Group P. Three patients were excluded because of losting to follow-up in Group PF. Finally, 97 patients in Group P and 101 patients in Group PF completed the full postoperative follow-up schedule (Fig. 2).

There was no significant difference in gender, age, BMI, BMD, the amount of bone cement injected, hospitalization time, VAS and ODI scores before the operation between the two groups(*P* > 0.05). Compared to Group P (34.36 ± 7.41), Operation time of Group PF (36.16 ± 11.34) was longer, but they showed no significant difference (*P* > 0.05). Compared with group P (41 patients), the number of patients who applied Cox-2 inhibitors within 3 days after operation was significantly fewer in group PF (9 patients) (*P* < 0.001). In Group P, bone cement leakage occurred in 5 cases, and adjacent segment fractures occurred in 2 cases. In group PF, 4 cases had bone cement leakage, and 3 cases had adjacent segment fractures. There was no significant difference in the complication such as pulmonary embolism, spinal cord injury, paraplegia in both groups (*P* > 0.05) (Table 1).

**Table 1:** Comparison of general data between Group P and Group PF

	Group P (*n* = 97)	Group PF(*n* = 101)	t/ (x^2^)	*P*
Male/female	41/56	44/57	(0.034)	0.854
Age (years)	77.17 ± 7.30	77.60 ± 8.25	−0.382	0.703
BMI (kg/m^2^)	24.38 ± 5.15	24.43 ± 5.01	0.076	0.892
BMD	−2.65 ± 0.47	−2.61 ± 0.43	−0.601	0.548
Hospitalization time (days)	4.16 ± 1.32	3.80 ± 2.31	1.859	0.065
Operation time (min)	34.36 ± 7.41	36.16 ± 11.34	−1.283	0.201
The number of patients who appliedCox-2 inhibitors within 3 days after operation	41	9	29.168	< 0.001**
The amount of bone cement injected (ml)	4.16 ± 1.04	4.21 ± 0.91	0. 632	0.715
VAS	7.56 ± 1.00	7.69 ± 1.07	0.741	0.410
ODI	69.45 ± 7.53	70.76 ± 6.68	0.937	0.362

Numeric data were expressed as Mean ± SD and analyzed by Independent-Samples T-test. Categorical data were expressed by the number of patients (%) and were analyzed with the χ2 test. Group P: PVP group; Group PF: PVP combined with FB group

*Abbreviations*: *BMI* body mass index, *VAS* visual analog scale, *BMD* bone mineral density, *PVP* percutaneous vertebroplasty, *FB* facet joint block

^**^P < 0.001, vs the two groups

### Comparison of VAS

In the two groups, the VAS score showed no difference before the operation and 3, 6 and 12 months after the operation (*P* > 0.05), the VAS score 1 day, 1, 3, 6, and 12 months after the operation showed significantly less compared to the pre-operation (*P* < 0.05). In Group PF, the VAS score 1 day (1.94 ± 1.12) and 1 month (2.08 ± 0.81) after the operation were significantly lower than that in Group P (3.16 ± 0.61) (2.81 ± 0.99) (*P* < 0.001) (*P* < 0.001) (Table 2).

**Table 2:** Comparison of VAS between Group P and Group PF at different time

Group	Pre-operation	Post-operation				
		1 day	1 month	3 months	6 months	12 months
Group P(*n* = 97)	7.56 ± 1.00	3.16 ± 0.61^#^	2.81 ± 0.99^#^	2.54 ± 0.96^#^	2.21 ± 0.88^#^	1.92 ± 1.05^#^
Group PF(*n* = 101)	7.69 ± 1.07	1.94 ± 1.12^#*^	2.08 ± 0.81^#*^	2.03 ± 0.77^#^	1.89 ± 0.74^#^	1.73 ± 0.65^#^
Time F, *P*	853.019,< 0.001					
Group F, *P*	56.703, < 0.001					
Time * Group F, *P*	4.763, < 0.001					

Data are presented as mean ± SD. The groups were compared by repeated measures analysis of variance (ANOVA). Bonferroni correction was used to correct multiple comparisons. Group P: PVP group; Group PF: PVP combined with FB group

*Abbreviations*: *VAS* visual analog scale, *PVP* percutaneous vertebroplasty, *FB* facet joint block

^#^*P* < 0.05, vs pre-operation in the same group; ^*^P < 0.05, vs Group P in the same time

### Comparison of ODI

In the two groups, the ODI score showed no difference before the operation and 6 and 12 months after the operation (*P* > 0.05), the ODI score 1 day, 1, 3, 6, and 12 months after the operation showed significantly less compared to the pre-operation (*P* < 0.05). In Group PF, the ODI score 1 day (41.27 ± 7.09), 1 month (39.58 ± 6.70) and 3 months (37.60 ± 4.88) after the operation was significantly lower than that in Group P (48.63 ± 7.51) (48.21 ± 8.66) (45.97 ± 5.83) (*P* < 0.001) (*P* < 0.001) (*P* < 0.05) (Table 3).

**Table 3:** Comparison of ODI between Group P and Group PF at different time

Group	Pre-operation	Post-operation				
		1 day	1 month	3 months	6 months	12 months
Group P (n = 97)	69.23 ± 6.94	48.63 ± 7.51^#^	48.21 ± 8.66^#^	45.97 ± 5.83^#^	36.46 ± 4.53^#^	36.18 ± 4.50^#^
Group PF(n = 101)	70.98 ± 6.85	41.27 ± 7.09^#*^	39.58 ± 6.70^#*^	37.60 ± 4.88^#*^	36.01 ± 5.59^#^	35.09 ± 3.86^#^
Time F, *P*	2139.084, < 0.001					
Group F, *P*	73.618, < 0.001					
Time * Group F, *P*	20.999, < 0.001					

Data are presented as mean ± SD. The groups were compared by repeated measures analysis of variance (ANOVA). Bonferroni correction was used to correct multiple comparisons. Group P: PVP group; Group PF: PVP combined with FB group

*Abbreviations*: *ODI* Oswestry Disability Index, *Group P* PVP group, *Group PF* PVP combined with FB group

^#^*P* < 0.05, vs pre-operation in the same group; ^*^P < 0.05, vs Group P in the same time

## Discussion

Osteoporosis is a progressive systemic disease, which often induces osteoporosis vertebral compression fractures. Recent studies have shown thatvertebroplasty can significantly reduce severe pain in acute OVCF patients within 6 weeks [8]. However, the incidence of residual pain in low back after PVP is not uncommon, with the lowest incidence of about 5% and the highest up to 22%, which seriously affects the postoperative quality of life of patients [6, 9–11].

The pain caused by OVCFs is mainly as a result of the fracture of the injured vertebra itself. Vertebroplasty (PVP) can reduce the micro motion of the fracture site and reshape spinal stability through the role of interface fixation [12]. Our study shows that the VAS scores for back pain and the ODI were significantly improved after the surgery in both groups (*P* < 0.05, respectively), and the VAS scores for back pain and the ODI in the PB group were significantly lower than those in the PKP group at 1 day and 1 month postoperatively.

In recent years, some scholars believe that the structure of the posterior appendage of the vertebral body is also an important source of pain [5], especially for the facet joints. In the elderly, the facet joints, muscles, ligaments and other tissues of the spine will degenerate, and fractures will further aggravate the above injuries [13, 14]. However, PVP can only resolve the pain caused by a vertebral fracture, it has a poor effect on pain relief caused by posterior spinal column injury, which may be the main cause of residual pain after PVP. In addition, the posterior medial branch of the lumbar spinal nerve is the only sensory innervation of the facet joints of the lumbar spine [15]. The posterior medial branch of the spinal nerve is run in a “bone fiber tube” at the junction of the upper edge of the transverse process of the lower vertebral body and the lateral edge of the superior articular process [16]. It is mainly distributed in the joint capsule, surrounded by abundant nerve endings. After vertebral compression fracture, the corresponding pathological changes appear in the posterior column of the spine, which stimulates the dorsal nerve branch and causes pain [17, 18]. The literature suggests that facet joint block, which can block the posterior medial branch of the spinal nerve, is effective in relieving the acute pain of vertebral compression fractures [19]. Compared with PVP, FB requires less expend and shows fewer complications, such as vein embolism and neural injury. Wang [20] presented a prospective randomized randomized controlled study, and in this study, they compared the pain relief in patients with osteoporotic vertebral compression fractures with the use of vertebroplasty or facet blocking. The results showed that PVP produced better pain relief than facet blocking in the short term, but in the long term the difference between these two techniques was insignificant. FB can’t restore vertebral height or reverse kyphotic deformity. Only one-third of patients technically suitable for vertebroplasty responded beneficially to FB in David’s research [21]. Kim et al. first investigated PVP and FB combined therapy and found that for OVCFs it was a profitable therapy [22]. As there are few studies to compare the efficacy of PVP and FB combined therapy with PVP alone. Thus, our study aims to compare the clinical outcomes of these two therapies.

According to this study’s follow-up results, we found that there were significant differences in VAS and ODI scores in the early postoperative period (immediately and 1 month after surgery) between the two groups. Since the VAS score 1 day after the operation in Group PF was significantly lower than that in Group P, the number of patients with Cox-2 inhibitors in Group PF decreased significantly within 3 days after operation. This is result confirmed that PVP combined with FB can provide better pain relief in OVCFs patients in short term. This is similar to that reported by Cheng et al. [23]. The pain associated with OVCFs may not only come from the vertebral body but also the posterior elements. PVP has a poor effect on pain relief caused by posterior spinal column injury, FB can eliminate pain originating from the posterior facet joint. Therefore, PVP combined with FB can both reduce the pain from vertebral body and from the posterior elements. It is suggested that facet joint block has a significant inhibitory effect on acute pain from the posterior elements. A possible reason is described as follows. Firstly, facet joint block has a definite effect on the posterior medial branch of thespinal nerve, and the analgesic effect is clear. Secondly, topical application of glucocorticoids can treat local aseptic inflammation after fracture and relieve pain. For fracture patients, the effect of surgery is the dominantfactor for whether the patient can perform early postoperative functional exercises, which is of positive significance for postoperative functional rehabilitation. This is one of the reasons why the ODI index and VAS score of the PVP combined with FB group is better than the PVP group. Our study found there was no significant difference in VAS and ODI scores in 6 months after the operation between the two groups. This may be related to the healing of vertebral fracture and the stability of the posterior elements for long time.

In our study, there was no significant difference in the mean operation time in both groups (*P* > 0.05). This is different from the report of Cheng et al. [23]. We think it is related to the different sequence of surgical steps. During waiting for the cement to solidify, we completed facet joint blocking, so the PVP combined facet joint block did not significantly extend the operation time. But it should be noted that the PF group has increased fluoroscopic exposure time, which should be informed to OVCFs patients before operation and weighed against better pain relief in the short term after the operation.

There were several limitations in the study. Firstly, the study was conducted in a small sample of patients. Secondly, the cases were from only one study center, larger clinical trials from more centers are needed for further study.

## Conclusion

The findings indicate that both PVP combined with FB and PVP alone are effective treatment methods for OVCFs. PVP combined with FB may be more effective and rapidly relieves acute back pain than PVP alone in the short term after the operation for the treatment of OVCFs. But these findings require confirmation with further studies.

## Data Availability

The authors will allow the sharing of participant data. The data will be available to anyone who wishes to access them for any purpose. The data will be accessible from immediately the following publication to 6 months after publication, and contact should be made via the first author by email.

## Abbreviations

OVCFs: Osteoporotic vertebral compression fractures
FB: Facet joint block
PVP: Percutaneous vertebroplasty
ASA: American Society of Anesthesiologists
VAS: Visual Analog Scale
ODI: Oswestry Disability Index
